# Productivity of the performance of visual perceptual tasks and symptom severity in patients with schizotypal disorder

**DOI:** 10.1192/j.eurpsy.2021.1402

**Published:** 2021-08-13

**Authors:** M. Vinogradova, A. Chepeliuk, O. Dorofeeva

**Affiliations:** 1 Department Of Neuro- And Pathopsychology, Faculty Of Psychology, Lomonosov Moscow State University, Moscow, Russian Federation; 2 Department Of Clinical Psychopharmacology, FSBI “Zakusov Institute of Pharmacology”, Moscow, Russian Federation

**Keywords:** schizotypal disorder, cognitive processes, visual perceptual tasks

## Abstract

**Introduction:**

The experimental research of visual perceptual processes in schizophrenia could shed a light on the psychological mechanisms of development of the illness.

**Objectives:**

To research the performance of visual perceptual tasks and its correlation with the symptom severity in patients with schizotypal disorder (SD).

**Methods:**

40 patients with SD in ICD-10 (mean age 29.8±8.3 years) were enrolled to the study. The Positive and Negative Symptoms Scale (PANSS) and two series of visual-perceptual tasks (Figures of Witkin and Goldstein) were applied. In series I subject should make a decision whether a complex figure contains a simple one without any feedback from the experimenter (all 96 trials). In series II each trial included two complex figures presented simultaneously (all 96 trials) that increased the visual-perceptual load. Statistical significance was ascertained by Spearman’s rank correlation.

**Results:**

Negative correlations were established between the number of right answers in series II of visual perceptual tasks and emotional withdrawal (r=-0.78, p≤0.01), passive/apathetic social withdrawal (r=-0.53, p≤0.05). Time of performance of series I and series II had negative correlations with preoccupation (r=-0.55 and r=-0.53, p≤0.05, respectively).

**Conclusions:**

The decrease in the productivity of visual perceptual tasks performance in case of additional load relates with reduced social and emotional dimensions of symptoms (social initiation, passivity, lack of sociality and inattention in daily activity, etc.) of patients with SD. Impulsivity in solutions (reduction of decision-making time) is associated with the increase of preoccupation with feelings, thoughts and autistic fantasies that lead to social and daily life disadaptation.

